# Motivational nondirective resonance breathing versus transcutaneous vagus nerve stimulation in the treatment of fibromyalgia: study protocol for a randomized controlled trial

**DOI:** 10.1186/s13063-020-04703-6

**Published:** 2020-09-23

**Authors:** Charles Ethan Paccione, Lien My Diep, Audun Stubhaug, Henrik Børsting Jacobsen

**Affiliations:** 1 Doctoral Fellow in Medicine and Health Sciences, Faculty of Medicine, University of Oslo, Klaus Torgårds 3, 0372 Oslo, Norway; 2 Department of Pain Management and Research, Oslo University Hospital, Ullevål, Kirkeveien 166, 0853 Oslo, Norway; 3 Oslo Center for Biostatistics and Epidemiology, Sognsvannsveien 9, 0372 Oslo, Norway

**Keywords:** Chronic widespread pain, Fibromyalgia, Heart rate variability, Pain intensity, Motivational nondirective resonance breathing, Vagus nerve stimulation

## Abstract

**Background:**

Chronic widespread pain (CWP), including fibromyalgia (FM), affects one in every ten adults and is one of the leading causes of sick leave and emotional distress. Due to an unclear etiology and a complex pathophysiology, FM is a condition with few, if any, effective and safe treatments. However, current research within the field of vagal nerve innervation suggests psychophysiological and electrical means by which FM may be treated. This study will investigate the efficacy of two different noninvasive vagal nerve stimulation techniques for the treatment of FM.

**Methods:**

The study will use a randomized, single-blind, sham-controlled design to investigate the treatment efficacy of motivational nondirective resonance breathing (MNRB™) and transcutaneous vagus nerve stimulation (Nemos® tVNS) on patients diagnosed with FM. Consenting FM patients (*N* = 112) who are referred to the Department of Pain Management and Research at Oslo University Hospital, in Oslo, Norway, will be randomized into one of four independent groups. Half of these participants (*N* = 56) will be randomized to either an experimental tVNS group or a sham tVNS group. The other half (*N* = 56) will be randomized to either an experimental MNRB group or a sham MNRB group. Both active and sham treatment interventions will be delivered twice per day at home, 15 min/morning and 15 min/evening, for a total duration of 2 weeks (14 days). Participants are invited to the clinic twice, once for pre- and once for post-intervention data collection. The primary outcome is changes in photoplethysmography-measured heart rate variability. Secondary outcomes include self-reported pain intensity on a numeric rating scale, changes in pain detection threshold, pain tolerance threshold, and pressure pain limit determined by computerized pressure cuff algometry, blood pressure, and health-related quality of life.

**Discussion:**

The described randomized controlled trial aims to compare the efficacy of two vagal nerve innervation interventions, MNRB and tVNS, on heart rate variability and pain intensity in patients suffering from FM. This project tests a new and potentially effective means of treating a major public and global health concern where prevalence is high, disability is severe, and treatment options are limited.

**Trial registration:**

ClinicalTrials.govNCT03180554. Registered on August 06, 2017.

## Background

Chronic widespread pain (CWP), including fibromyalgia (FM), is one of the most difficult chronic pain conditions to successfully treat [1]. CWP is characterized by long-lasting pain that persists for longer than 3 months in multiple regions of the body and is commonly associated with a variety of psychophysiological symptoms such as fatigue, cognitive impairments, and psychological distress [2]. Available CWP/FM treatments provide only modest improvements in pain and minimum improvements in both physical and emotional functioning [3]. Opioids fail to alleviate pain intensity and function [4] and cause a myriad of adverse side-effects [5] while complementary and alternative treatments have only weak to moderate effect sizes for treating CWP [6]. However, vagal nerve innervation may provide us with innovative and successful opportunities to target the complex psychophysiological framework of FM [7–9].

Preliminary intervention trials on humans [10, 11] have shown that vagus nerve stimulation (VNS) can modulate multiple pathophysiological mechanisms inherent in various CWP conditions such as FM: VNS has shown to strongly reduce peripheral inflammatory cytokines [12] decrease sympathetic tone [13], decrease oxidative stress [14], and reverse pain-related brain activity patterns [7, 15]. To date, VNS has been traditionally administered through invasive procedures, known as invasive VNS (iVNS), which typically involves the surgical implantation of electrodes around the vagus nerve [9]. However, iVNS has a high risk for adverse events [16] that often requires removal of the iVNS device. An effective noninvasive alternative to iVNS is transcutaneous VNS (tVNS). The tVNS system sends electrical impulses safely through the skin of the outer ear straight into the auricular branch of the vagus nerve [17]. Another noninvasive approach of vagal stimulation could be through contemplative-based practices and respiratory means.

Various forms of paced slow breathing have also shown to influence brain electrical activity which may be mediated by VNS arising from the diaphragm [18]. This cardiorespiratory stimulation of the vagus nerve may explain some of the overall positive emotional and cognitive benefits of diaphragmatic breathing (DB) [19]. However, the positive analgesic effects deep breathing may have on some acute pain conditions has failed to be established for CP conditions such as CWP [8]. In particular, research on mindfulness-based meditation interventions show contradictory findings [20], differences in conceptualization and practice [21], positive report biases [22], and only small to moderate effect sizes for treating pain in clinical populations [7, 23, 24]. Experimental evidence elucidating the underlying psychophysiological mechanisms of how deep breathing may be used to treat CWP is lacking and often inconsistent [8]. DB as a means of VNS may potentially decrease the pathophysiological processes involved in central sensitization as seen in FM. This action may be the mechanism by which VNS reduces widespread musculoskeletal pain in FM and other comparable pathologies [9].

Due to the strong bidirectional relationship between pain, respiration, and the vagus nerve, a recent systematic review [8] called for future research to identify the autonomic and cardiovascular mediators that link respiration and pain, identify the physiological mechanisms needed to reduce pain, identify the central mechanisms responsible for producing hypoalgesia, and identify the psychological (i.e., behavioral) mechanisms needed to reduce pain. New approaches to testing the efficacy of delivering noninvasive VNS for pain as well as designing new contemplative-based approaches that can potentially optimize vagal tone in order to treat those with CP are needed [7].

## Methods/design

### Aim

The primary aim of this protocol article is to describe the design of a randomized controlled clinical trial investigating the effects of motivational nondirective resonance breathing (MNRB) and transcutaneous vagus nerve stimulation (tVNS) on photoplethysmography (PPG) measured heart rate variability (HRV) in patients diagnosed with FM. Secondary outcomes are changes in self-report numeric rating scale (NRS) pain intensity, pain detection threshold (PDT), pain tolerance threshold (PTT), and pressure pain limit (PPL) determined by computerized cuff-pressure algometry (CPA), blood pressure (BP), and health-related quality of life. The principal objective is to explore the following four research questions:

1. Does a standardized sham-controlled tVNS intervention or MNRB intervention have effects upon PPG- measured HRV?
2. Is a substantial change in HRV associated with a significant change of self-report NRS pain intensity?
3. Is a substantial change in HRV associated with a change in computerized cuff-pressure algometry PDT, PTT, and/or PPL?
4. Are there changes in BP following either an active or sham tVNS intervention and/or a MNRB intervention?
5. Are there effects on health-related quality of life and behavior following either an active or sham tVNS intervention and/or a MNRB intervention?

### Design

**Fig. 1:**
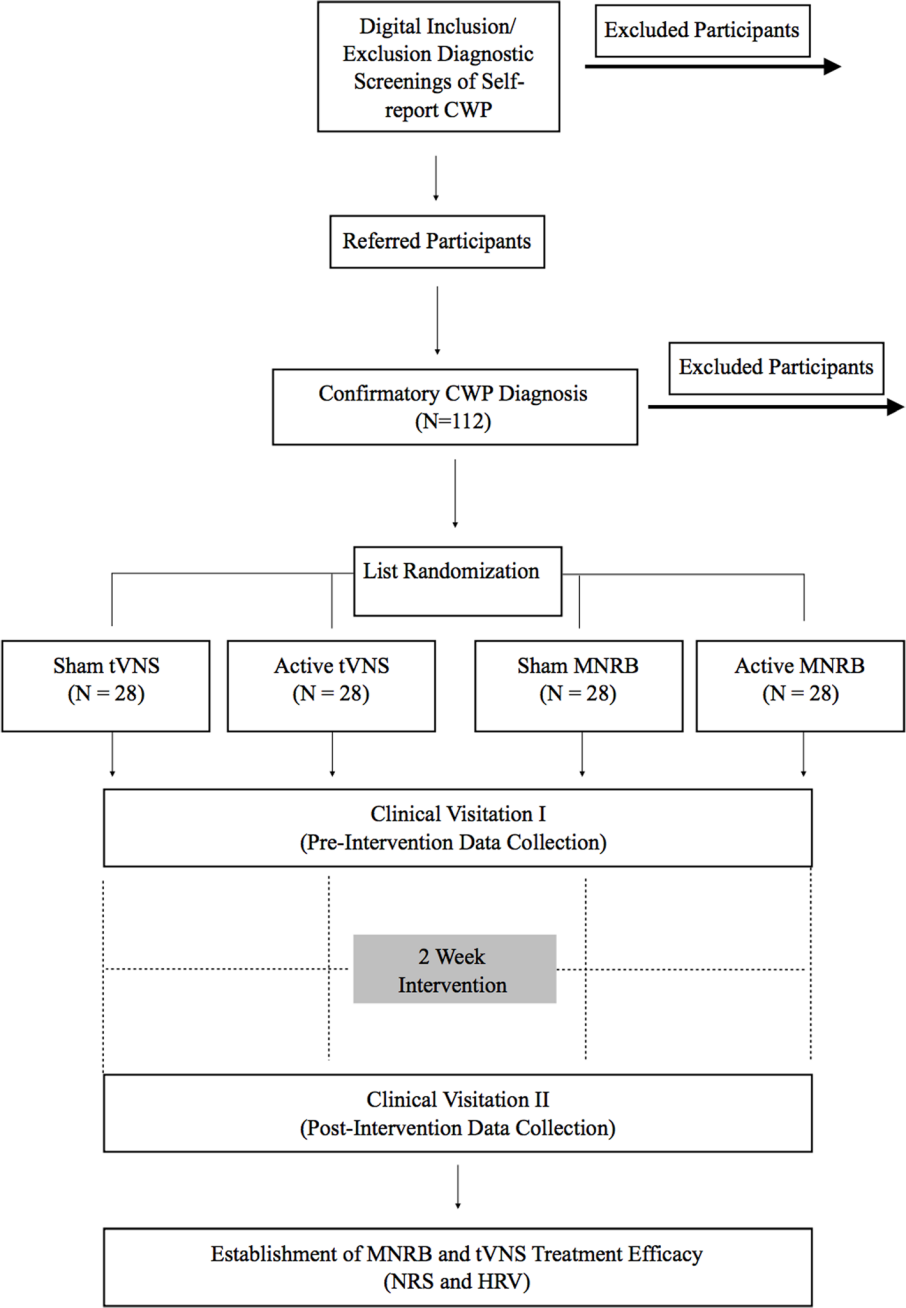
Overview of study design

This study will use a single-blind randomized controlled experimental design which will be reported according to the CONSORT statement [25] and the Guidelines for Reporting Articles on Psychiatry and Heart rate variability (GRAPH) [26] in order to expedite translational research efforts and improve research methods, replication, and peer review [27–29] (Table 1). A total of *N* = 112 consenting FM patients will be consecutively recruited and randomized from the Department of Pain Management and Research at Oslo University Hospital, Ullevål, in Oslo, Norway, during the Summer and Fall of 2019. Participants will be randomized to either an experimental tVNS group, a sham tVNS group, an experimental MNRB group, or a sham MNRB group. Both active and sham treatment interventions will be delivered at home, twice a day, for 15 min in the morning and for 15 min in the evening, for a total duration of 2 weeks (14 days). Treatment adherence to both interventions will be monitored electronically through a portable Android device and from a Daily Treatment Journal. An 80% completion of tVNS stimulation and MNRB training (a completion of 23 treatment sessions out of a total 28) will be regarded as adequate adherence in this project. Participants are invited to the clinic twice for pre- and post-intervention data collection. An overview of the participant selection, study design, and study flow is illustrated in Fig. 1.

**Table 1:** World Health Organization Trial Registration Data

Data category	Information
Primary registry and trial identifying number	ClinicalTrials.gov NCT03180554
Date of registry in primary registry	08/06/2017
Secondary identifying numbers	Southeast Regional Health Authority, Norway, Project Number: 2017/766 Cristin Project ID: 619480
Source of monetary and material support	Southeast Regional Health Authority, Norway
Primary sponsor	Southeast Regional Health Authority, Norway
Contact for public queries	Charles Ethan Paccione, M.S., M.A., Ph.D. Fellow Email: charlespaccione@gmail.com
Contact for scientific queries	Charles Ethan Paccione, M.S., M.A., Ph.D. Fellow Email: charlespaccione@gmail.com Department of Pain Management and Research Oslo University Hospital, Ullevål
Public title	Body versus Machine: Meditative Breathing versus Vagus Nerve Stimulation in the Treatment of Chronic Widespread Pain
Scientific title	Body versus Machine: Motivational Nondirective Resonance Breathing versus Transcutaneous Vagus Nerve Stimulation in the Treatment of Fibromyalgia
Country of recruitment	Norway
Health condition studied	Chronic widespread pain, fibromyalgia
Interventions	Motivational nondirective resonance breathing (active and Sham) Transcutaneous vagus nerve stimulation (active and sham)
Key inclusion and exclusion criteria	Inclusion criteria: • Confirmatory diagnosis of chronic widespread pain, including fibromyalgia; widespread pain index (WPI) ≥ 7 and symptom severity scale (SSS) score ≥ 5 OR WPI of 4–6 and SSS score ≥ 9; generalized pain in at least 4 of 5 body regions must be present; pain symptoms have been generally present for at least 3 months; average pain intensity ≥ 6 on a 0–10 numerical rating scale, where 0 represents “no pain” and 10 represents the “worst pain imaginable” Exclusion criteria: • History and/or presence of comorbid severe neurological or psychiatric disorders (e.g., mania, psychosis, suicidality, bipolar/schizophrenia/autism spectrum disorders); neurodegenerative disorders (e.g., Parkinson’s, Alzheimer’s, Huntington’s disease); pregnancy or planned pregnancy; planned surgery; eating disorder (e.g., obesity, anorexia nervosa); head trauma; migraine; active heart implants (e.g., pacemaker); active ear implants (e.g., cochlear implant); individuals who have practiced meditation consistently (for more than 20 min/day) within the last 6 months
Study type	Randomized controlled clinical trial Interventional Allocation: randomized Intervention model: parallel assignment Masking: double blind (subject, caregiver, investigator, outcomes assessor) Primary purpose: treatment
Date of first enrolment	June 6, 2019
Target sample size	112
Recruitment status	Recruiting
Primary outcome	Heart rate variability (HRV)
Key secondary outcome	Numerical rating scale for average pain intensity; pain detection threshold; pain tolerance threshold; pressure pain limit; blood pressure; credibility/expectancy; health-related quality of life; stress and depression; interoceptive awareness; spirituality; catastrophizing; interference

### Covariates

Sociodemographic characteristics which can have a significant impact upon HRV include age [30], sex [31], body mass index (BMI), waist-to-hip ratio (WHR), physical activity levels [32], usual sleeping habits and hours slept prior to clinical visitation, meal consumption within 2 h prior to clinical visitation, oral contraceptive use for women, habitual levels of alcohol [33], nicotine [34], and caffeine intake. Cardiovascular diseases [35], psychophysiological disorders such as depression and anxiety [36], as well as various cardioactive medications have a considerable impact upon HRV and pain and will be accounted for. In addition, antidepressant classes (e.g., tricyclics) [37], some antipsychotic classes (e.g., clozapine) [38], benzodiazepines [39], antihypertensives [40], some types of statins, and some prescription pain medication use may significantly affect HRV and will be recorded. Non-prescription pain medication as well as sleep aids will also be documented.

### Inclusion criteria

Participants must be between the ages of 18 and 65 with an average NRS of 6–10 [41] and must have a confirmatory diagnosis of chronic widespread pain (CWP), including fibromyalgia (FM), (Read Code: MG30.01) as defined in the ICD-11 [42]. CWP is currently defined by the American College of Rheumatology 2010/2011 criteria to be a fundamental feature of FM, defined as pain lasting ≥ 3 months, located axially, above and below the waist, and on both sides of the body with physical symptoms that include fatigue and waking unrefreshed [43]. This study will use the 2016 revision to the 2010/2011 FM diagnostic criteria [44] which introduces important changes based on experience both within clinical and research settings. A participant included in this study must satisfy the following 3 conditions:

1. Widespread pain index (WPI) ≥ 7 and symptom severity scale (SSS) score ≥ 5 OR WPI of 4–6 and SSS score ≥ 9.
2. Generalized pain, defined as pain in at least 4 of 5 regions, must be present. Jaw, chest, and abdominal pain are not included in generalized pain definition.
3. Symptoms have been generally present for at least 3 months.
4. A diagnosis of fibromyalgia is valid irrespective of other diagnoses. A diagnosis of fibromyalgia does not exclude the presence of other clinically important illnesses [44].

### Exclusion criteria

Participants must not have any past history and/or presence of comorbid severe neurological or psychiatric disorders (e.g., mania, psychosis, suicidality, bipolar/schizophrenia/autism spectrum disorders) [26] and/or neurodegenerative disorders (e.g., Parkinson’s, Alzheimer’s, Huntington’s disease). Participants will be further excluded on pregnancy or planned pregnancy [45]; planned surgery; receiving treatment for any type of eating disorder (e.g., obesity, anorexia nervosa, etc.) [46]; head trauma; migraine; active heart implants (e.g., pacemaker) [47]; and active ear implants (e.g., cochlear implant). Individuals who have practiced meditation consistently (for more than 20 min/day) within the last 6 months will also be excluded [48].

### Enrolment procedure

Study and contact information will be posted and updated regularly on the Oslo University Hospital website, ClinicalTrials.gov, CRISITN (Current Research Information System In Norway), and various social media platforms. After reading about the study, interested participants are instructed to log in to Nettskjema—a secure digital data management and collection system in Norway—and fill out a brief digital inclusion/exclusion form consisting of exclusion criteria, diagnostic criterion for FM, and an NRS scale. If participants meet self-reported inclusion criteria, they will be contacted by testing administrators with an invitation to participate and an appointment day and time for both clinical visitation I (CVI) and clinical visitation II (CVII) at the Department of Pain Management and Research, Oslo University Hospital.

Upon arrival at the Department for CVI, participants are provided with an informed consent. At this time, participants will have the opportunity to ask any of the testing administrators questions about the study and their contribution. Once the informed consent is signed, participants will be formally enrolled into the study.

### Data management

This study will use Viedoc—an electronic data capture web-based solution used for clinical trial data collection and management that complies with all relevant health regulations [49] and the FDA Code of Federal Regulations 21 Part 11. The Clinical Trial Unit at Oslo University Hospital will perform the setup and design of our Viedoc electronic case report form (eCRF) and audit the study flow throughout the trial period. Testing administrators will actively use Viedoc for all participant data entry throughout the entirety of the study. Signature of the testing administrator is reacquired to ensure the completeness and accuracy of the participant data that has been obtained in the eCRF.

Data omissions and/or corrections in Viedoc will be justified and accounted for within the eCRFs. After the database is locked, the investigator (CEP) will receive a digital copy of the subject data for archiving at the investigation site. The data will be securely stored at the research site database at Oslo University Hospital. Data will be de-identified so that each study participant is only recognizable by his/her unique Viedoc trial subject number. The data will be stored 5 years for further analyses and voluntary follow-up 6 months, 1 year, and 3 years after study closure.

### Randomization and blinding procedure

#### Randomization

The computer-generated randomized allocation sequencer will be imported into the Viedoc eCRF system and made available to testing administrators. Only testing administrators will have access to participant treatment allocation and the allocation will not be available until the participant has signed the informed consent and deemed eligible to participate in the study.

This investigation will use a list randomization recruitment method [50] for CVI where participants will be randomly allocated to receive one of the four treatment groups: tVNS #1 (active); tVNS #2 (sham); MNRB #1 (active); MNRB #2 (sham). The randomization will be stratified by sex (male = 1, female = 2) and cardioactive medications (yes = 1, no = 0) with varying block size within strata. Viedoc will generate the randomization number as well as the allocated treatment group for each participant.

Participants will be considered as taking cardioactive medication if they report that they are currently taking any medication for high BP, cholesterol, heart disease, or prescription pain medication. Participants will also be considered as taking cardioactive medication if they answer that they use tranquilizers, antidepressants, and/or antipsychotics either “less than every week,” “every week, but not daily,” or “daily.” Participants are permitted to continue taking any previously prescribed pain medications/psychopharmacological treatments that are necessary during the trial period.

#### Blinding

The blinding strategy utilized in this clinical trial follows guidelines previously set forth and designed specifically for non-pharmaceutical intervention trials [51]. As recommended [52], research design elements such as treatment type, active and inactive treatment mechanisms, and research hypotheses are concealed from participants, testing administrators, and data collectors. Active and sham treatment allocation is concealed from the participants and testing administrators. Both testing administrators and study participants will be told that they will provide/receive two different *versions* of nerve stimulation at different locations on the ear (for the tVNS group) or that there are two breathing techniques that are being explored (for the MNRB group) in this investigation [53]. The testing administrators will introduce either “Version 1” (active) or “Version 2” (sham) of the treatment interventions.

The principal investigators (CEP and HBJ) are blinded to patient treatment allocation as well as the randomization form in Viedoc. This form will only be visible to the testing administrators performing participant data collection. To protect against export of blinded data during the study, there will be two export roles in this study: (1) blinded export and (2) unblinded export. Blinded data export role will be given to the investigators prior to database lock while the unblinded data export role will be given to the investigators only after database lock.

#### Statistical considerations and sample size calculation

An independent trial statistician who is blinded to the treatment allocation will complete the initial analysis for the main outcomes. Data analyses will be performed using IBM SPSS version 25, R software, and SAS® software after importing data from Viedoc. To sufficiently detect a difference between groups in HRV as it relates to NRS pain intensity, a sample size between 30 and 77 (depending on the HRV metric used) is typically needed [54]. However, subgroups are commonly employed within designs that have been suggested to require 20 participants per cell [55]. Furthermore, researchers have typically used Cohen’s calculations of small (0.2), medium (0.5), and large (0.8) effect sizes when quantifying the magnitude of group differences for HRV investigations. However, it has been recommended that these guidelines should only be used when the effect size distribution (ESD) is unknown; analyses have shown that Cohen’s guidelines underestimate the magnitude of small and large effect sizes and that HRV studies are generally underpowered [56]. A change on the NRS of 20% as our secondary outcome measure in this study for participants between CVI and CVII will be considered to be a clinically significant treatment efficacy [57, 58].

Due to our power calculation and in light of these findings, effect sizes of 0.25, 0.5, and 0.9 should be interpreted as small, medium, and large effects (after rounding to the closest 0.05). To achieve a statistical power of 0.8 to detect a large effect size, 21 participants are required per group in a case-control study [56]. However, in order to account for possible participant dropout, 28 participants per group will be included which correlates to a statistical power of 0.9. This recommended sample size which is based upon the aforementioned ESD can be tailored to our specific study used to appropriately power this research investigation [56]. This makes it more likely to better replicate and derive true effect size estimates. Mean with standard deviation or median with interquartile will be reported for continuous variables/data. Frequency and percentage will be given for categorical variables/data.

Missing data will be treated as follows: If the first (morning pre-intervention) NRS/HRV recording is missing, the last (evening post-intervention) NRS/HRV recording will be used from the night before. If the second (morning post- intervention) NRS/HRV recording is missing, then the first (morning pre-intervention) NRS/HRV recording will be used. If the third (evening pre-intervention) NRS/HRV recording is missing, then the first (morning pre- intervention) NRS/HRV recording will be used. Finally, if the last (evening post-intervention) NRS/HRV recording is missing, then the third (evening pre-intervention) NRS/HRV recording will be used. If all morning and evening NRS/HRV recordings are missing from a day, then NRS/HRV recordings from the day before will be used based upon the assumption that improvement/change is not present. The patient-reported outcome measures will be modeled by repeated measures and fitting random effects models with random slopes. Random effects models are considered the gold standard when analyzing repeated measures and handling missing data in longitudinal design [59, 60].

Baseline characteristics as mean with standard deviation or median with interquartile and frequency with percentage will be described for the four participant groups, active and sham. The change from the first to second data points will be calculated for the 14-day treatment session in regard to HRV and NRS present pain intensity. The change from CVI to CVII HRV, NRS present pain intensity, and NRS average pain intensity will be determined. Difference in changes between the groups in the morning session and evening session will be examined and tested by using two-way analysis of variance (ANOVAs) or random intercept and slope models. For overall difference in the changes between the four groups regardless of morning, evening or days, random intercept models or repeated one-way analysis of variance (ANOVAs) will be used to analyze the data. The tests will be two-sided, and the significance level will be set to 0.05.

#### Description of intervention arms

##### Motivational Nondirective Resonance Breathing™ (MNRB™)

MNRB™ is a meditation-based deep breathing intervention developed by the lead author (CEP) [7] which is based on emergent findings in integrative neuroscience and autonomic self-regulation in cardiopulmonology.

Participants will perform MNRB™ at home for 15 min [61] in the morning upon waking and 15 min at night before going to bed (preferably at the same time for each individual participant) for a total duration of 2 weeks. Participants will use the BarTek™ device in order to practice MNRB™. The BarTek™ device is a CE-approved respiratory gating device compatible with an Android smartphone. Krüger&Matz Flow 5 Android smartphones will be used for running and recording the MNRB™ program with the BarTek™ respiratory gating device when practicing either active or sham MNRB™. Participants will receive MNRB™ user training at CVI and be provided with a BarTek™ operational worksheet which they will follow every morning and evening when practicing MNRB™.

(A) *Active MNRB™* will be practiced relaxed, sitting back in a chair no more than 30° from the horizontal, with both feet flat on the floor, hands on thighs with palms facing downward. Participants are further instructed to not talk or make any movements during their treatment session. The BarTek™ respiratory gating device is placed upon the diaphragm—around the abdomen, below the rib cage, and an inch (about two finger widths) above the navel. Participants open the MNRB™ program on the Krüger&Matz Flow 5 Android phone, which is connected wirelessly via Bluetooth to the BarTek™ respiratory gating device, and are guided through a 15 min MNRB™ breathing intervention which guides participants from an average respiration rate of 12 breadths/min to a resonance frequency rate of 6 breadths/min—the most optimal means of increasing cardiac-vagal tone (HRV) via respiration [47]. Out of a full (100%) breathing cycle, participants are instructed to use the stomach to breathe in to full inspiratory capacity for 30% and exhale for 60% by tightening and pulling the stomach back toward the spine. At the end of each inspiration and expiration, participants are instructed to retain their breadth for 5% of the cycle [62]. The participant is instructed to allow the chest to remain immobile throughout the entirety of the session [63, 64]. This is achieved through a feedback system actively engaging the patient to follow an orb that indicates stages of breathing and constantly correcting participants when they practice. While practicing, participants are to engage in a nondirective state of mind [65], where a relaxed focus of attention is established by listening to the inspiration and expiration sound guides of the MNRB™ program. Attention is neither directed toward staying with the respiration sound guides nor directed toward observing the spontaneous flow of thoughts and sensations [65]. Sensations, such as pain, during MNRB™ are accepted without actively directing attention toward them or away from them [7].

(B) *Sham MNRB™* is practiced relaxed, sitting back in a chair no more than 30° from the horizontal, with both feet flat on the floor, hands on thighs with palms facing upward. Participants are instructed not to talk or make any movements during their treatment session. Participants will be instructed to breathe at the normal respiration rate for an adult (12 breadths/min) [66] by following a respiratory pacer [67] on the Krüger&Matz Flow 5 Android MNRB™ program while counting their breadth [53]. Out of a full (100%) breathing cycle, participants are instructed to breathe normally with a 49% inhale and a 49% exhale without any (i.e., 1%) breath retention at the end of each inspiration and expiration. Participants are instructed to maintain a focused attention on their breath while actively detecting mind wandering [68].

##### Transcutaneous vagus nerve stimulation (tVNS)

**Fig. 2:**
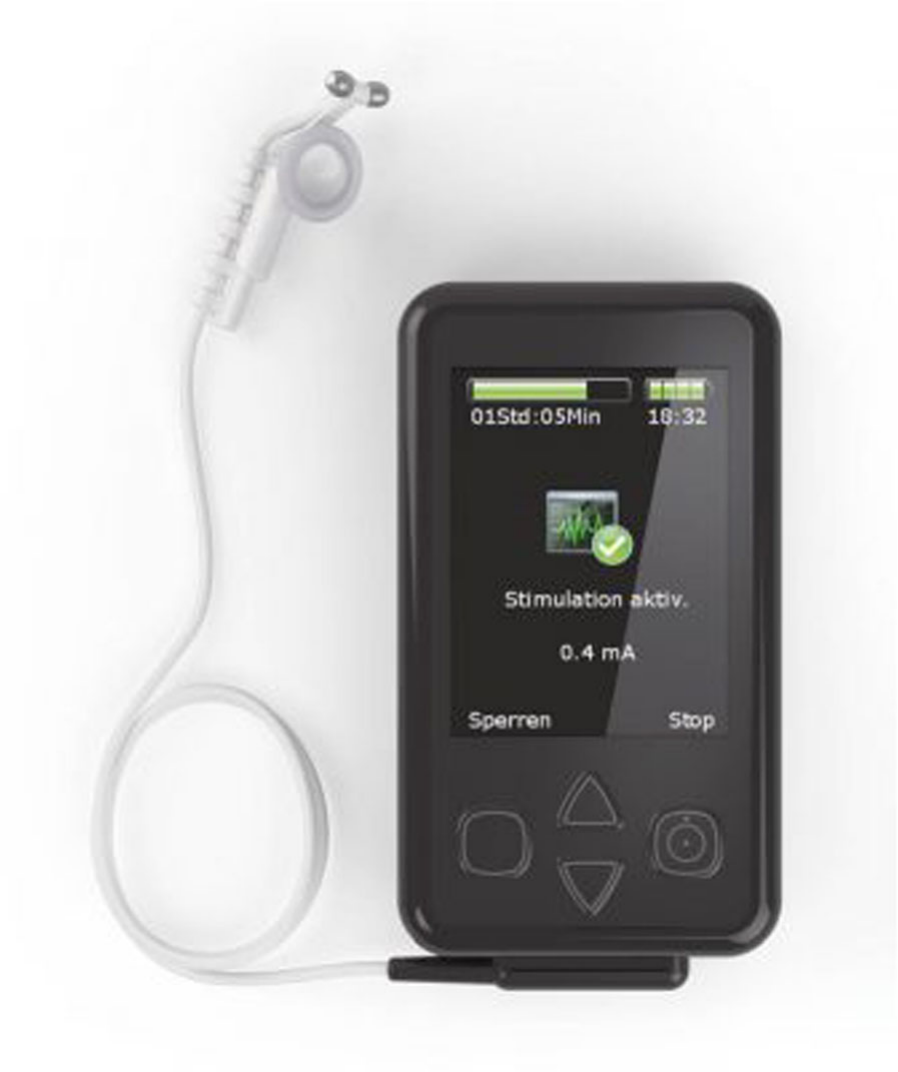
tVNS device. Nemos® transcutaneous vagus nerve stimulation (tVNS) device (Figure taken from [69])

The tVNS device (“Nemos®”; cerbomed GmbH, Erlangen, Germany) (Fig. 2) stimulates the afferent auricular branch of the vagus nerve located medial of the tragus at the entry of the acoustic meatus [70]. This device has received CE approval as indication that it complies with essential health and safety requirements [71]. The ear is first cleaned with an alcohol wipe and the electrode is sprayed with a conductive fluid to ensure optimal stimulation. Two titan electrodes mounted on a gel frame are connected to the Nemos® pocket-size stimulator and placed in the concha of the left ear in order to avoid stimulation of fibers to the heart. Stimulation intensity is individually adjusted (from 0.1 to 10 mA) with a pulse width of 250 μs and a consistent stimulation frequency of 25 Hz for optimal stimulation [72]. During CVI, participants will familiarize themselves with the stimulation device and its proper usage under the guidance of the testing administrator. During this visitation, the intensity of the tVNS will be slowly increased until the optimal intensity (mA) is reached (i.e., a slightly uncomfortable tingling sensation) for each individual participant [53]. Both active and sham stimulation constantly alternate between active stimulation for 30 s, followed by a break of 30 s [73]. Participants will perform active or sham tVNS at home for 15 min [61] in the morning upon waking and 15 min at night before going to bed (preferably at the same time for each individual participant) for a total duration of 2 weeks. Due to habitation, participants will be allowed to readjust this stimulation intensity during their 2-week intervention period if needed.

(A) *Active tVNS* is performed in a relaxed position, sitting back in a chair no more than 30° from the horizontal, with both feet flat on the floor, and hands on thighs with palms facing downward. The bipolar stimulation electrode is placed correctly within the concha of the left ear. Participants are instructed to breathe normally while not talking or making any movements during their session.
(B) *Sham tVNS* is performed in a relaxed position, sitting back in a chair no more than 30° from the horizontal, with both feet flat on the floor, and hands on thighs with palms facing upward. The bipolar stimulation electrode is turned 180° and placed incorrectly over the center of the left earlobe instead of the outer auditory canal [15]. This area is known to be free of cutaneous vagal innervation [17] and produces no activation in the cortex and brain stem [15]. Participants are instructed to breathe normally while not talking or making any movements during their session.

### Data acquisition

Testing administrators input data directly into Viedoc on a Windows 7 HP EliteDesk 800 G2 SFF desktop computer located in the clinical visitation room at the Department of Pain Management and Research, Oslo University Hospital, Ullevål. Height is recorded with a Seca 206 (Seca GmbH, Hamburg, Germany) device which is bolted into the wall and leveled. Weight is obtained using an ADE M320000 (ADE Germany GmbH, Hamburg, Germany) digital electronic floor scale. Waist and hip circumferences are recorded with a MyoTape (AccuFitness, LLC, Greenwood Village, USA), and BP is taken utilizing a Philips IntelliVue MMS X2 (Philips Medizin Systeme GmbH, Hamburg, Germany) multi-measurement module and transport monitor.

#### CameraHRV

**Fig. 3:**
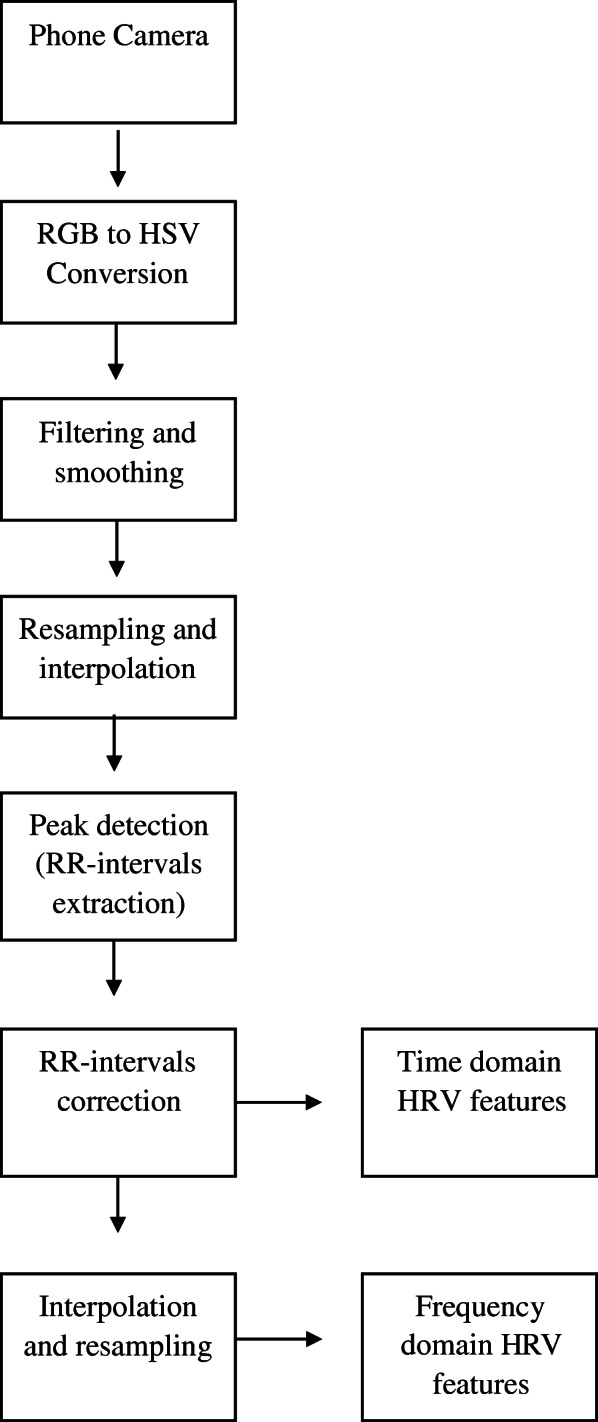
Signal processing pipeline for CameraHRV (Table taken from [80])

Photoplethysmography (PPG)-measured HRV data will be obtained from CameraHRV (Marco Altini, Amsterdam, Netherlands)—an Android App which has been utilized in multiple clinical trials [74–76] and validated with both the Polar H7 device and the golden standard electrocardiography (ECG) [77]. PPG-measured HRV is a reliable means of computing HRV [78] and will be used to assess heart rate as well as time-based (AVNN, standard deviation of NN intervals (SDNN), root mean square of successive differences (rMSSD), percentage of successive normal sinus RR intervals more than 50 ms (pNN50)) and frequency-based (LF, HF) resting HRV. Krüger&Matz Flow 5 Android smartphones without SIM card and telecommunication capability will be used to run CameraHRV and record HRV for both the clinical visitations and the patient daily readings. HRV values will be computed via the reflection through the illumination of the skin of a participant’s right index finger using the Krüger&Matz Flow 5 camera’s flash. CameraHRV detects the amount of light that is reflected by the camera located next to the light source [79] (see Fig. 3 for signal processing).

This study will use resting HRV recordings of 1 min. Reducing the HRV recording window to a duration of 1 min, in comparison to the standardized 5-min recording, is acceptable when rMSSD is considered as the primary HRV parameter of interest [81]. A 1-min recording of the natural log of rMSSD (lnRMSSD) has also been proven to offer good reliability in comparison to the classical 5-min recording of rMSSD [82]. Furthermore, high-frequency HRV (hfHRV) also shows reasonable agreement between ultrashort-term recording windows (60 s or less) and 5-min periods [82, 83]. Several studies use hfHRV as an index of vagal tone [47] due to its strong correlation with rMSSD [84, 85]. However, unlike hfHRV, rMSSD has been shown to be insusceptible to confounding respiratory effects during the recording window [26, 83]. In addition to the main analysis performed with one ideal variable reflecting vagal tone (RMSSD), it is recommended [47] that researchers perform the same analyses with the other variables depicting vagal tone (hfHRV, SDNN, and pNN50). Date and time of day for every HRV recording will be saved within the CameraHRV program which can be used as a proxy for treatment compliancy for all participants during their 2-week home treatment.

#### BarTek™ (Fig. 4)

The BarTek™ respiratory gating device (VRMind, Wroclaw, Poland) designed for practicing MNRB™ measures abdominal expansion via strap tension that is induced by the diaphragm during the entire respiratory cycle. The elastic strap of the BarTek™ sensor is placed around the waist of each participant and is adjusted in length in order to produce a minimal resistance to respiratory movement. Tension measurement is implemented by a strain gauge circuit. This circuit contains a strain gauge measurement element, and a temperature compensation element. High-precision measurement is ensured by using a high-resolution analog digital converter. Device output signal has an electric potential difference that is sampled at 80 Hz. The signal is transmitted to the Krüger&Matz Flow 5 Android smartphone via a Bluetooth Low Energy protocol. Date and time of day along with all respiratory information will be recorded and saved within the MNRB™ program. The raw signal processing used in order to obtain each participant’s respiration dynamics (e.g., rate and volume) is shown in Fig. 5.

**Fig. 4:**
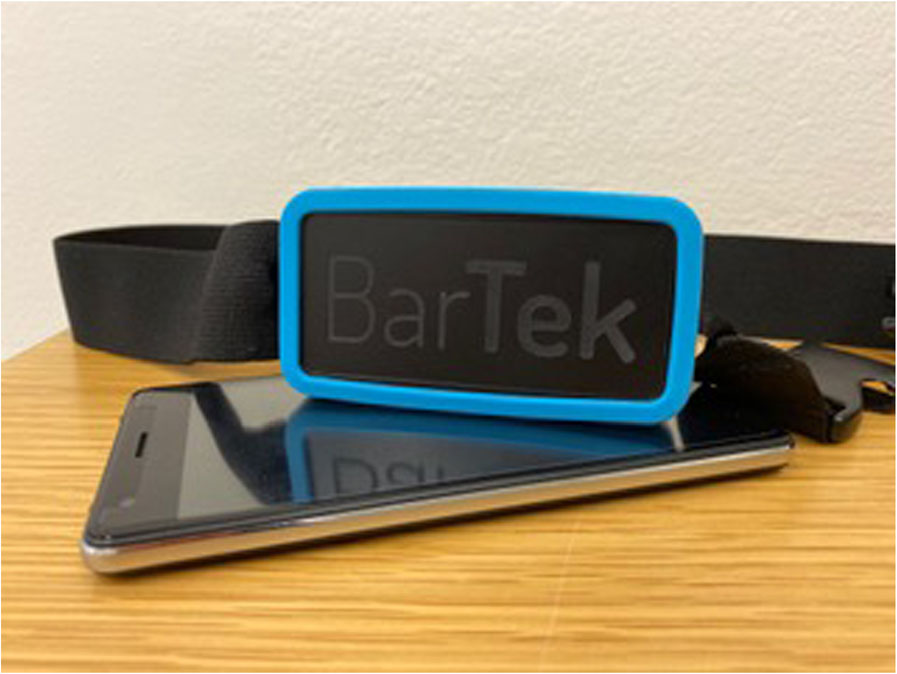
BarTek design

**Fig. 5:**
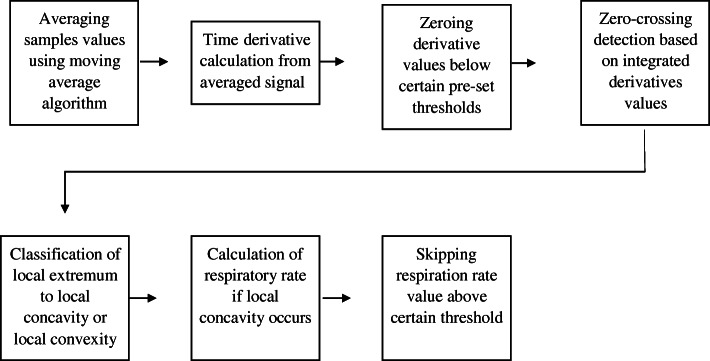
Signal processing pipeline for BarTek device. Real-time signal processing is based on a zero-crossing algorithm with further modifications. It is important to note that zero-crossing detection is based on a dynamically adjusted threshold. The basic signal processing algorithm consists of seven steps

#### DoloCuff

Computerized cuff-pressure algometry (CPA) will be administered using the DoloCuff device with software version 2.0.5.1 (DoloCuff; Unique Electronic Aps, Hvidovre, Denmark) in order to assess clinical pain sensitivity mechanisms in FM patients. The DoloCuff CPA consists of a double-chambered 13-cm-wide textile high-pressure 13.5 cm × 76 cm tourniquet cuff (VBM Medizintechnik GmbH, Sulz, Germany), a computer-controlled air compressor (Unique Electronic Alps), a 10-cm electronic visual analog scale (VAS), and a stop button for immediate release of air in the tourniquet cuff. The tourniquet is tightly mounted around the widest part of the m. gastrocnemius in order ensure reliable pressure readings.

Ramp inflation of 1 kPa/s [86] will be used in order to record a participant’s pain detection threshold (PDT), pain tolerance threshold (PTT), pressure pain limit (PPL), and stop time [87]. Cuff PDT is defined as the pressure value the first time the VAS score exceeds 0 (i.e., at the moment of transition from a sensation of strong pressure to first sensation of pain) whereas cuff PTT is defined as the pressure value when the participant terminates the pressure inflation (i.e., when the pain due to the pressure of the cuff becomes intolerable) [87]. A maximum pressure of 150 kPa and a maximum time under pressure of 180 s are set as the upper limits throughout the study [88]. The maximum pain intensity (VAS peak) and time to VAS peak will be extracted along with the individual slopes in pain intensity rise and fall from the start of cuff inflation to the end of cuff inflation. Areas under the VAS curve are also calculated based on raw data [89].

#### ViedocMe

Participant-reported outcome measures (PROMs) in the form of questionnaires (see below) will be completed electronically using the ViedocMe functionality available in Viedoc at CVI and CVII. Study staff will create a ViedocMe account for each participant in the participant’s Clinic View in Viedoc and provide a unique log-in profile (username, pin code, and ViedocMe web address) for each participant. Participants are to use this information to log in to their personal ViedocMe account on a tablet (Samsung Galaxy Tab A6 32GB) which is connected to Oslo University Hospital’s secure wireless account. The questionnaires in ViedocMe will only be available for completion the day of each clinic visitation. Participants will complete the questionnaires at the end of each visitation. After the time-window has expired (i.e., at midnight on the day of the clinic visit), questionnaires can no longer be completed electronically. If problems arise using ViedocMe, or if the participant prefers to use paper forms, paper copies of the questionnaires will be handed out to the participant and the data will be entered into Viedoc by testing administrators. Questionnaires are only available to participants in Norwegian. For a list of names with descriptions of each questionnaire utilized in this study, see the Appendix.

List of questionnaires in the order of their completion:

1. Credibility Expectancy Questionnaire
2. Participant Global Impression of Change
3. EQ-5D-5L
4. Hopkins Symptom Checklist
5. Multidimensional Assessment of Interoceptive Awareness, version 2
6. Spiritual and Religious Attitudes in Dealing with Illness
7. Pain catastrophizing scale
8. Brief Pain Inventory
9. Insomnia Sleep Inventory
10. Oswestry Low Back Pain Disability Questionnaire
11. General Health Questionnaires

#### Daily treatment journal

Participants will receive a take-home Daily Treatment Journal where they will record their treatment session day, time (morning or evening), pre- and post-treatment HRV recording (Yes/ No), and pre- and post-treatment NRS pain intensity. Participants will also be instructed to write any thoughts, feelings, and/or reflections in regard to their overall treatment experience. Upon completion of the 2-week treatment, participants will hand their Daily Treatment Journal to the assigned research administrator at CVII where it will be recorded directly into Viedoc.

### Data collection procedure

#### Clinical visitations I and II (Fig. 6)

Testing administrators will conduct all data collection for CVI and CVII at the Department of Pain Management and Research under the supervision of the principal investigator. All data will be collected by the same assessor throughout each visitation. From 8:00 to 12:30, three participants will be consecutively registered upon arrival at the Department and directed to the clinical visitation room by an assigned testing administrator. Upon entering the visitation room, each participant will be given an informed consent and will be offered the time to ask any questions in regard to what is required of them for their participation. Upon signing the informed consent, measurements of height, weight, BMI, and WHR will be made. Participants will be instructed to remove any heavy clothing and footwear when on the digital electronic floor scale. BMI will be calculated as weight in kilograms divided by the square of the height in meters (kg/m^2^). After recording the aforementioned measurements, participants will sit in a relaxed semi-Fowler position (30° tilt from the horizontal) with feet flat on the floor, hands on thighs, and palms facing upward for the remainder of the clinical visitation. Any discrepancies to the procedure will be logged in Viedoc and kept for the remainder of the study period and will be included as a potential confounder in analyses.

**Fig. 6:**
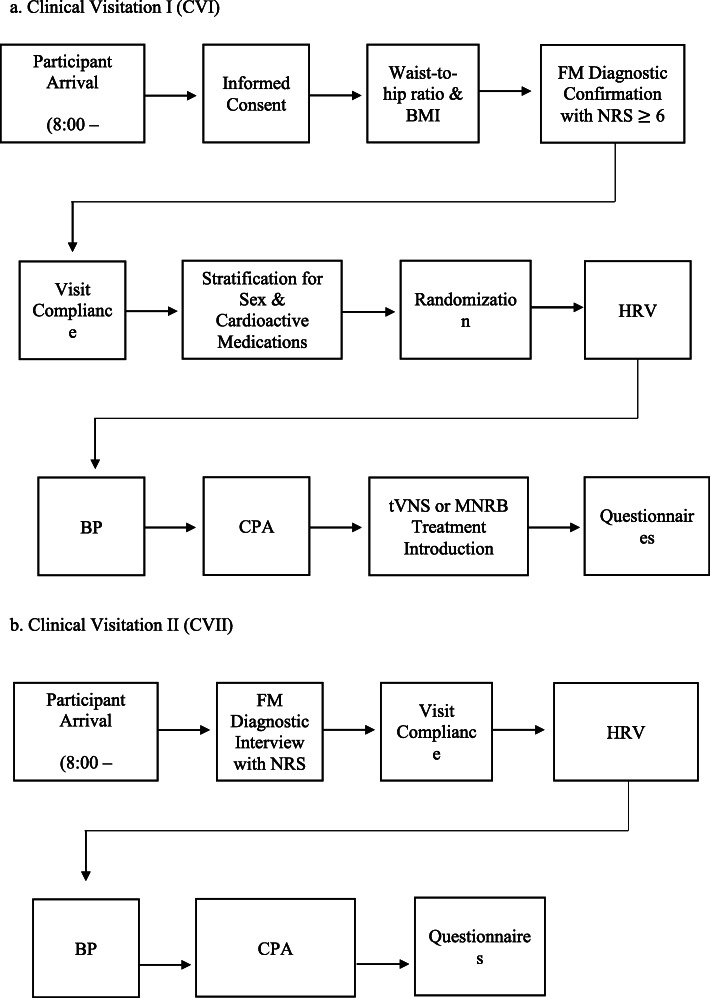
Clinical visitations. **a** Clinical visitation 1 (CVI). **b** Clinical visitation II (CVII)

The assigned testing administrator will then conduct a clinical interview at CVI in order to confirm a diagnosis of FM, including FM, and in CVII in order to determine how or if the pain has changed. Participants will be guided through an electronic version of the 2016 revision to the 2010/2011 FM diagnostic criteria form [44] digitalized in Viedoc by the lead author (CEP). Prior to the FM diagnostic confirmation interview, participants will be asked to numerically rate their pain in the following manner: “On a scale of 0 to 10, where 0 corresponds to no pain and 10 corresponds to the worst possible pain you can imagine; (1) How strong would you say that the pain usually is?; and (2) How strong is the pain now?” If participants are confirmed to have FM with an average (i.e., usual) pain intensity NRS ≥ 6, they are instructed to remain seated in a semi-Fowler position (no more than 30° from the horizontal), with eyes closed, and breathe normally without speaking or making any movements [47]. For CVII, there are no diagnostic criteria cutoffs for FM and average pain intensity.

The testing administrator will then conduct a visit compliance interview immediately following the FM diagnostic interview for each visitation. Participants are instructed prior to visitation to follow a normal sleep routine [90] and abstain from any type of intense physical training the day before each clinical visitation [91]. Participants are also asked to abstain from drinking coffee, any type of energizing drinks [92], and tea [93] 2 h before each clinical visitation. Furthermore, participants are instructed not to drink alcohol 24 h prior to their visitations [94] or eat within 2 h of their visitation [95] (eating/digestion [96], missing a meal [97], or smoking [34] can significantly influence HRV). If patients do partake in the aforementioned actions, this information will be accounted for at this time. Upon completion of the visit compliance interview, testing administrators will note the sex of each participant and any cardioactive medications that they are taking. The computer-generated randomized allocation sequencer in Viedoc then generate the randomization number as well as the allocated treatment group for each participant (e.g., tVNS #1 (active); tVNS #2 (sham); MNRB™ #1 (active); MNRB™ #2 (sham)).

**Fig. 7:**
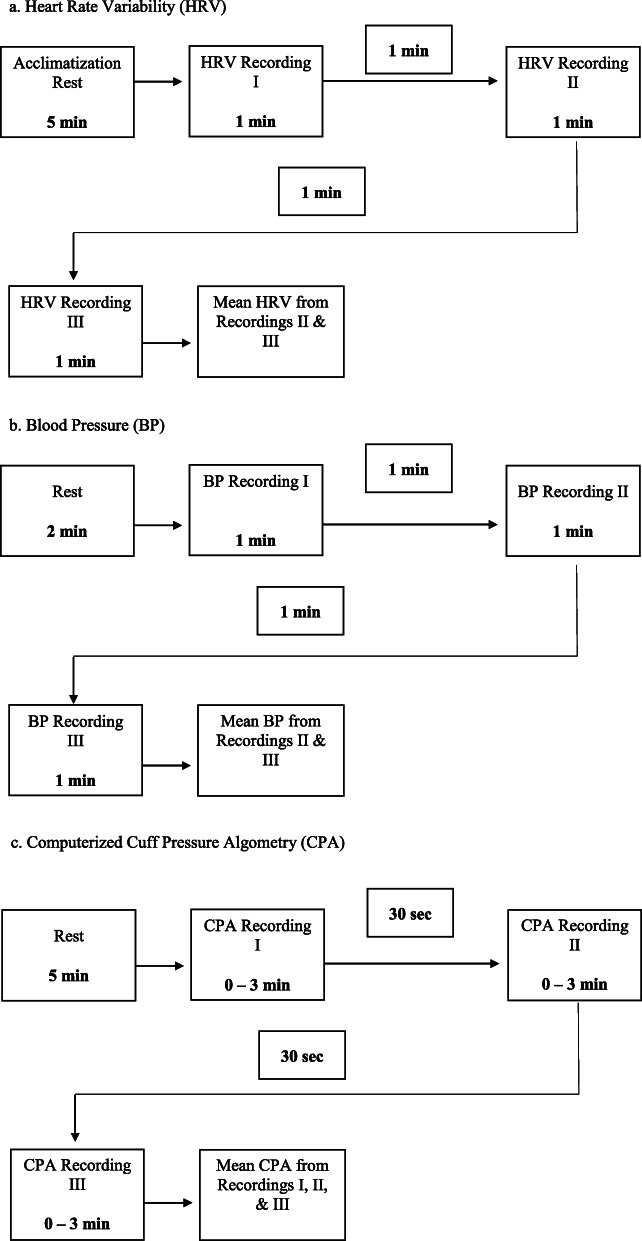
Data collection timeline. **a** Heart rate variability. **b** Blood pressure. **c** Computerized cuff-pressure algometry

In order to acclimatize to the HRV recording environment, each participant is instructed to remain seated for at least 5 min [98] prior to taking the first HRV measurement (Fig. 7a). Acclimatization helps reduce HRV changes due to posture changes [99, 100] and can also reduce confounds subsequent to participant test anxiety [26]. Immediately following the 5 min acclimatization period, three 1-min HRV recordings will be taken on the tip of the right index finger of each participant separated by 1-min intervals. The beginning and end of each of the three HRV recordings will not be announced due to the impact of attentive states and test anxiety on respiratory frequency [101] and HRV recordings [102]. An average of the last two recordings will be used as the baseline measure.

At least 2 min following the three HRV recordings, resting systolic, diastolic, and mean blood pressure (BP) will be measured (Fig. 7b). The correct size cuff is chosen after the circumference of the upper arm is determined. Three readings on the upper right arm are taken separated by 1-min intervals. The average of the last two measurements will be used in the analyses [103]. This BP assessment protocol is similar to that used in several population studies [104–106].

Following the BP assessment, CPA will be administered (Fig. 7c). Participants are asked to continuously rate perceived pain if the sensation of pain increases, decreases, or remains the same, by using the electronic VAS module which is placed in their dominant hand. The VAS ranges from 0-cm (no pain) to 10-cm (worst pain imaginable). Furthermore, participants are instructed to press a red stop button located directly below their VAS console if and when the sensation of pain becomes intolerable at any time. The testing administrator conducting the data collection will also have the ability to stop the exercise at any moment if needed via the computer. Three CPA sessions will be recorded on each leg individually: first the left leg, then the right leg, and finally the left leg, each separated by a 30-s interval. The average of all three sessions will be used in the final analyses.

For clinical visitation I, each participant will be introduced to their assigned treatment (tVNS or MNRB™) and version (version 1 or 2). Testing administrators are to motivate each participant to complete the treatment and provide instructions on how to use the devices throughout the 2-week intervention period. In addition to a verbal introduction of each treatment version, testing administrators will also guide participants in a short 5-min trial period during which the treatment is properly setup and tested. This is helpful in demonstrating to participants the functionality and design of their treatment. Participants are welcome to ask any questions or share any concerns in regard to the procedure and what is demanded of them at this time. When the treatment introduction and trial period is complete for CVI, participants will receive their Daily Treatment Journal (or return it at CVII) and complete questionnaires in their ViedocMe account (For assessments as a function of timepoints according to the 2013 SPIRIT guidelines, see Table 2). Patients who receive one version of the treatment will be voluntarily offered the second version 6 months after the end of the study if they are still in pain and the results of the study justify this.

**Table 2:** Assessments as a function of timepoints (according to the 2013 SPIRIT figure guidelines)

Measure	Target	T0	Intervention	T1
		*Baseline*	*Morning and evening treatment (4 recordings/day)*	*Post-intervention (2 weeks)*
NRS	Average pain intensity	X		X
NRS	Current pain intensity	X	X	X
HRV	Hear rate variability	X	X	X
BMI and WHR	Body mass index and waist-to-hip ratio	X		
BP	Blood pressure	X		X
CPA	Experimental pain threshold, tolerance, and limit	X		X
CEQ	Treatment efficacy expectation	X		
PGIC	Treatment efficacy impression			X
EQ-5D-5L	Health-related quality of life	X		X
HSCL-25	Stress and depression	X		X
MAIA-2	Interoceptive awareness	X		X
SpREUK-15	Spirituality	X		X
PCS	Catastrophizing	X		X
BPI	Pain interference	X		X
ISI	Insomnia	X		X
ODI	Functional disability	X		X
General health	Overall health/nutrition and sociodemographic	X		

#### Daily intervention procedure (Fig. 8)

Upon waking or going to sleep, participants are to take a 1-min HRV recording with CameraHRV before and after their assigned 15-min treatment intervention. Participants are instructed to use the same HRV recording procedure as in CVI and CVII: sitting in a relaxed semi-Fowler position no more than 30° from the horizontal, knees at a 90° angle, while breathing normally, with both feet flat on the floor without moving or talking. Due to the fact that circadian rhythms and digestion have an impact upon HRV [107, 108], time of day and time since last meal should be standardized when possible in short-term HRV recordings, especially for designs incorporating repeated recordings over time [26]. Therefore, participants are instructed to take their HRV recordings preferably at the same time every day in the morning and at night before and after their assigned intervention. Furthermore, participants are instructed to keep a normal sleep schedule throughout their 2-week intervention while abstaining from consuming any large meals, caffeinated drinks, nicotine, or alcohol immediately prior to their morning HRV recordings and no less than 2 h before their evening HRV recordings. HRV recordings will be saved locally on the assigned Krüger&Matz Flow 5 phone and labeled with the participant’s assigned Viedoc identification number. Participants are to legibly encircle their NRS after each 1-min HRV recording (four times per day) in their Daily Treatment Journal throughout the 2-week intervention period.

**Fig. 8:**
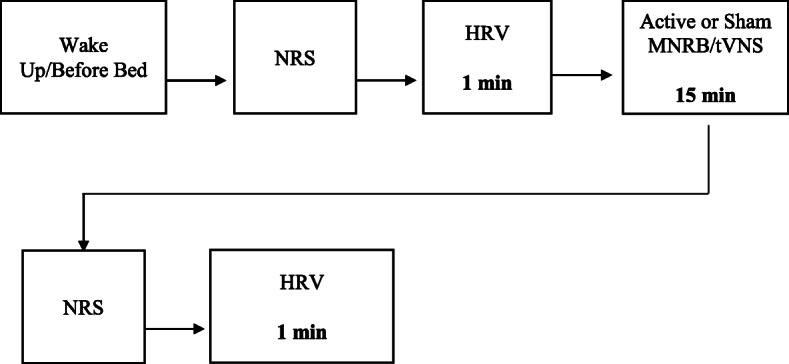
Daily home intervention procedure

## Discussion

CWP, including FM, affects one in ten individuals within the general population [109] and has the highest prevalence in Norway (12–30%) [110] where it is a leading cause of long-term sick leave and disability [111]. Due to an overall lack of efficacy in both mainstream [112] and alternative [6] treatments, it is necessary to develop new avenues for treatment.

### Strengths

A recent systematic review [8] called for future research to identify the autonomic/cardiovascular mediators that link respiration and pain; identify the physiological (i.e., respiratory) mechanisms needed to reduce pain; identify the central mechanisms responsible for producing respiratory hypoalgesia; and identify the psychological (i.e., behavioral) mechanisms needed to reduce pain. This clinical trial will answer that call by investigating whether the autonomic mediator that links respiration and FM is HRV; the physiological mechanism needed to reduce pain in those with FM is a 30-5-60-5 diaphragmatic breathing (i.e., resonance frequency breathing) technique; the central mechanism responsible for producing respiratory hypoalgesia is vagus nerve stimulation (either through respiratory or electrical means); and the cognitive and affective psychological mechanisms needed to reduce pain in those with FM is nondirective attention and motivation.

Meditative-based breathing techniques commonly investigated in clinical trials are often poorly described [113–115] and do not account for the relative frequency of diaphragmatic movement with precision, nor does it account for effects of expectation and attention on pain outcomes [8]. MNRB™ will be delivered to participants with specific instructions in regard to breathing frequency, volume, and breathing mechanics. Monitoring the participants’ breathing with the BarTek™ respiratory belt will show how well patients have complied with these instructions and help identify pain-related compensatory changes in breathing depth and frequency. Participant treatment expectation will be accounted for through the Credibility/Expectancy Questionnaire (CEQ) prior to the start of their active or sham MNRB™ intervention. And attention/distraction will be controlled for by giving the sham-control group a sham MNRB™ technique of counting the breath.

Vagus nerve innervation as a means of pain treatment has been traditionally administered through invasive procedures, known as invasive vagus nerve stimulation (iVNS), which has typically involved the surgical implantation of electrodes around the cervical vagus nerve [116]. iVNS is often accompanied with a high risk for adverse events (e.g., voice alteration, paresthesia, cough, headache, dyspnea, pharyngitis, and pain at the site of stimulation) [72]. This study will deliver transcutaneous VNS (tVNS) as a safe and effective alternative which allows the user to directly modulate stimulation intensity, pulse duration, and frequency accordingly [117]. Both means of vagal stimulation (MNRB™ and tVNS) are easy to use, portable, and safe and are able to be tailored to a patient’s stimulation or respiratory threshold. These factors increase the probability for patient compliancy and overall satisfaction with the treatment interventions.

Treatment compliance is heavily dependent upon treatment duration. Most researchers have traditionally assumed that meditation practice has its effects in a cumulative way through long-term practice. However, current research [118] shows that short-term influences of meditation practice have a more promising effect upon clinical outcomes. Continual meditation practice may not be necessary for maintaining beneficial psychophysiological effects [119]. This study shares this notion by employing a mind-body intervention which lasts only 2 weeks (as compared to the traditional 8-week mindfulness program) and practiced for only 30 min in total each day. Shortening the typical daily practice duration of mind-body interventions [68] as well as practicing in a natural setting [120] increases participant compliancy and satisfaction.

### Limitations

Within-subject designs are typically recommended over between-subject designs for HRV experiments—unlike between-subject designs, within-subject designs offer optimal experimental control and reduce the impact of external factors such as medication, alcohol, and smoking [102]. However, within-subject designs for HRV research also suffer from many inherent weaknesses (such as the learning effect that can be observed in some experimental tasks [47]. If a between-subject design is chosen with testing occurring on different days, it is recommended that participants take part in the experiment at the same time of the day [107]—a practice which has been implemented in this current study.

As mentioned in previous studies investigating pain and respiration [121, 122], not blinding the experimenters to the intervention can be seen as a limitation. Even though it is typically considered unfeasible to blind the experimenter to the intervention for investigations of this kind, it has been recommended [123] that studies document expectation bias by carefully designing and standardizing the intervention instructions and measuring participants’ expectations about the effectiveness of the to-be-delivered intervention—both of which are exercised in this study. Nonetheless, the difficulty in designing a reliable sham breathing protocol for experimental and clinical trials is quite apparent—trials that implement sham breathing protocols commonly instruct participants to either “breathe normally” [53] or “spontaneously breathe” [124] while “focusing on their breadth” [125]. Unlike a pharmacological intervention, a behavioral therapy is not easily controlled and participants can potentially know which intervention they are receiving [51]. This is especially true for trials employing deep breathing exercises due to the fact that the sham, normal paced breathing, is known to be commonly associated with resting and not with active treatment. Longitudinal research [126] comparing daily variations of time and frequency-based HRV parameters between controlled breathing (i.e., pacer breathing) and spontaneous breathing (i.e., natural breathing) sessions found that significant time-based HRV correlations exist between these two different conditions (especially in regard to rMSSD). This demonstrates that during a longitudinal follow-up, these markers provide the same HRV variations regardless of breathing pattern.

Even though a number of studies using high-intensity tVNS have not found any major side effects, as mentioned before, tVNS can still be accompanied by slight pain, burning, tingling, or itching sensations near the sight of the electrodes [72]. There is also no scientific consensus regarding the frequency and strength of tVNS stimulation for pain treatment [9] nor is there a clear understanding of how a constant pulse frequency mirrors endogenous vagal nerve activity—the vagus nerve most likely does not communicate/activate in regimented 30-s consecutive intervals as most of the tVNS devices do [127].

### Impact and dissemination of results

The knowledge generated from this investigation will inform patients as well as policy makers and healthcare providers within the field of pain research and management. If shown to have a significant effect upon HRV and pain intensity, tVNS stimulators and/or MNR™ treatment and BarTek devices will be investigated further and be provided to FM patients as reliable, noninvasive, and effective means of treatment. Currently, tVNS devices have a narrow patient distribution while the MNRB™ program and accompanying BarTek device have never been evaluated before. The portable nature of these devices, their easy user ability, and the time needed to receive each treatment can increase patient compliance and autonomy while easing the treatment burden of healthcare providers.

These findings will be published in open-access peer-reviewed journals in order to help inform existing treatment procedures and guide the development of new integrative pain treatment programs at both public and private sector clinics. Trial results will also be presented at international and national conferences for both healthcare providers and patients. The main results of this study will be published in 2020/21.

### Trial status

At the time of this manuscript submission, participant recruitment is ongoing. The current protocol version is in accordance with two ethics approval amendments.

Protocol Version: 3

Date: 4/27/2020

Recruitment Start Date: 6/5/2019

Recruitment End Date: 8/1/2020

## Data Availability

Unidentifiable participant data from this trial will be available to researchers who provide a methodologically sound proposal of interest and who fulfill institutional guidelines. All of the individual participant data collected during the trial will be available after de-identification, beginning 9 months and lasting 5 years after publication. Requestors must sign a data access agreement form with Oslo University Hospital.

## Appendix

### Participant-reported outcome measures (PROMS)

#### Numeric rating scale

The numeric rating scale (NRS) [128] will be used to assess pain intensity. Participants are asked to circle a number between 0 and 10 that best describes their pain intensity—zero indicates “no pain at all” whereas the upper limit represents “the worst pain ever possible.” The NRS is a self-administered, public domain scale [128] that describes a less-subtle distinction of pain levels unlike the VAS/GRS, where there are an unlimited number of possible answers [129]. However, as in VAS/GRS, a change on the NRS of 20% between two timepoints of an assessment is considered clinically significant [57, 58]. Numerical rating scales correlate highly with other pain assessment tools [130, 131] and have good compliance [58] and reliability. In addition to clinical visitations I and II, participants are to complete the NRS pre and post MNRB/tVNS intervention every day (four times per day) and record their response in their Participant Journal.

#### Credibility/Expectancy Questionnaire

The Credibility/Expectancy Questionnaire (CEQ) is a self-report measure of a participant’s expectations about the efficacy of a particular treatment and whether they think that the treatment is credible or not. In particular, it investigates two factors—what one feels and what one thinks in regard to the treatment. The CEQ is composed of six items which are scored on a 9-point scale ranging from “not at all logical,” “somewhat logical,” and “very logical.” Items 4 and 6 ask the participant how they feel and how they think the administered treatment will improve their overall health state in regard to their pain on a 0–100% scale, where 0% represents “no improvement” whereas 100% represents “total improvement.” The CEQ will only be delivered during clinical visitation I.

#### Patient Global Impression of Change

The Patient Global Impression of Change (PGIC) is a self-report measure of a patient’s belief about the efficacy of a treatment and their overall improvement. Patients rate their change on a 7-point scale: “very much improved,” “much improved,” “minimally improved,” “no change,” “minimally worse,” “much worse,” or “very much worse” [132]. Despite a weak positive correlation between PGIC and improvement in standard FM/CWP outcome measures, the PGIC is still believed to be an important source of patient-reported symptoms and a clinically relevant tool in the assessment of perceived impact of FM disease management [133]. The PGIC is especially helpful when paired with other domain-specific questionnaires and/or items following interdisciplinary psychologically based treatments for pain [134]. The PGIC will only be delivered during clinical visitation II.

#### EQ-5D-5L

The EQ-5D-5L is a standardized instrument developed by the EuroQol Group to measure health-related quality of life. The EQ-5D consists of a descriptive system and the EQ visual analog scale (EQ VAS). The descriptive system comprises five dimensions: mobility, self-care, usual activities, pain/discomfort and anxiety/depression. Each of these dimensions has 5 levels which include the following: “no problems,” “slight problems,” “moderate problems,” “severe problems,” and “extreme problems.” The patient indicates their health state by ticking a box next to the most appropriate statement in each of the five dimensions. This results in a score that expresses the level selected for that dimension. The digits for the five dimensions are then combined into a 5-digit number that describes the patient’s health state. The EQ VAS uses a vertical visual analog scale to record the patient’s self-rated health. The endpoints on the EQ VAS are labeled “The best health you can imagine” and “The worst health you can imagine.” The VAS is used as a quantitative measure of health outcome that reflects the patient’s own judgment. The EQ-5D-5L has been translated and validated in Norwegian [135].

#### The Hopkins Symptom Checklist-25

The Hopkins Symptom Checklist-25 (HSCL-25) [136] will be used to assess emotional distress, anxiety, and depressive symptoms. The HSCL-25 scale consists of 25 questions about the presence and intensity of the most common psychiatric symptoms of anxiety and depression. Participants are asked: “To what extent have you been bothered by the following symptoms in the last 14 days including today?” Responses include the following: 1 (not at all), 2 (a little), 3 (quite a bit) and 4 (extremely). The HSCL-25 has reliable sensitivity and specificity and has been extensively used and tested in a Norwegian population [137] with both external and internal validity [138].

#### Multidimensional Assessment of Interoceptive Awareness

The Multidimensional Assessment of Interoceptive Awareness (MAIA-2) [139] will be used to assess participants’ interoceptive body awareness and, in particular, provide pertinent information in regard to how emotions and the perception of pain are related to interoception. The MAIA is a 32-item multidimensional instrument comprising of eight scales (e.g., noticing, non-distracting, not-worrying, attention regulation, emotional awareness, self-regulation, body listening, and trusting) ranging from 3 to 7 items each. Each of these eight scales includes items that either are duplicates or are similar to items previously published: MAIA items 1, 6, 18, 20, and 27 are derived from the Scale of Body Connection, Bodily Dissociation Subscale, MAIA item 29 from the Multidimensional Body-Self Relations Questionnaire, MAIA item 5 from the Mindful Attention Awareness Scale and MAIA item 4 from the Kentucky Inventory of Mindfulness Skills [140]. Due to the fact that interoception is considered a significant mediator of therapies developed for CP conditions, the MAIA scale will help investigate intervention-related changes in body awareness to the clinical outcomes under investigation [141].

#### SPREUK-15 Spirituality Questionnaire

The SpREUK-15 investigates whether or not participants rely on spirituality as a resource to cope with pain. It investigates three factors: (1) having trust/faith; (2) search for a transcendent source to rely on; and (3) reflection of life and subsequent change of life and behavior. Items are scored on a 5-point scale from disagreement to agreement (0—does not apply at all; 1—does not truly apply; 2—do not know (neither yes nor no); 3—applies quite a bit; 4—applies very much). The scores can be referred to a 100% level (transformed scale score). Scores > 50% indicate higher agreement (positive attitude), while scores < 50 indicate disagreement (negative attitude).

#### Pain Catastrophizing Scale

The Pain Catastrophizing Scale (PCS) [142] will be used to assess pain catastrophizing. The PCS contains 13 items rated on a 5-point scale from 0 (not at all) to 4 (all the time). The total score for the PCS ranges between 0 and 52, with a higher score demonstrating more severe catastrophizing [143]. A total PCS score of 30 represents a clinically relevant level of catastrophizing and corresponds to the 75th percentile of the distribution of PCS scores in clinical samples of CP patients [144]. The PCS is valuable for addressing intervention efficacy because it has been studied extensively across many different CP populations and is commonly used as a key outcome in determining the success of interventions that target CP.

#### Brief Pain Inventory- Pain Interference Scale

The Brief Pain Inventory (BPI) [145] will be used to assess pain interference. As one of the standard psychometric tools for clinical trials of pain [146, 147], the BPI provides two subscales: pain interference and pain severity. Due to the fact that the NRS will be utilized to assess pain intensity in this study, only the BPI pain interference scale will be used. Pain interference (seven items) is rated on a 0–10 scale, where 0 indicates “no interference” whereas 10 indicates “complete interference.” There are no clinical cutoff scores for the BPI, but the arithmetic mean of the seven interference items can be used as a measure of pain interference. This mean can be used if more than 50%, or four of seven, of the total items have been completed upon administration [145].

#### Insomnia Severity Index- Items 1, 2, and 3

The Insomnia Severity Index (ISI) is a brief 7-item self-report questionnaire which will be used to assess the nature and severity of both nighttime and daytime components of insomnia [148]. The usual recall period is the “last month” and the dimensions evaluated are as follows: severity of sleep onset, sleep maintenance, and early morning awakening problems, sleep dissatisfaction, interference of sleep difficulties with daytime functioning, noticeability of sleep problems by others, and distress caused by the sleep difficulties. A 5-point Likert scale is used to rate each item (e.g., 0 = no problem; 4 = very severe problem), yielding a total score ranging from 0 to 28. The total score is interpreted as follows: absence of insomnia (0–7); sub-threshold insomnia (8–14); moderate insomnia (15–21); and severe insomnia (22–28) [149]. It is available in several languages and is increasingly used as a metric of treatment response in clinical research—the ISI is a reliable and valid instrument to detect cases of insomnia in the population and is sensitive to treatment response in clinical patients [149].

#### Oswestry Low Back Pain Disability Questionnaire

The Oswestry Disability Index (ODI—also known as the Oswestry Low Back Pain Disability Questionnaire) [150] evaluates a patient’s permanent functional disability. The test is considered the “gold standard” of low back functional outcome tools. It is designed to investigate how pain is affecting a subject’s ability to manage in everyday life. There are 10 sections which include the following: pain intensity, personal care, lifting, walking, sitting, standing, sleeping, sex life, social life, and traveling. Each section has six statements which the subject is instructed to choose dependent upon how accurate each statement is for describing their current situation. For each section, the total possible score is 5: if the first statement is marked the section score = 0; if the last statement is marked, it = 5.

#### General Health

The General Health questionnaire [151] evaluates a participant’s cohabitation, overall physical activity intensity and frequency, overall alcohol and tobacco use, overall caffeine intake, oral contraceptive use for women, importance of religion (e.g., very important, somewhat important, or not important), and types of treatments received for pain (i.e., surgery, physiotherapy, acupuncture, and complementary and alternative medicine). These questions are taken from the Tromsø 6 population study (2007–2008) in Norway and have been clinically validated and delivered to 12,984 men and women 30–87 years of age.

## Abbreviations

CVI/CVII: Clinical visitation I/clinical visitation II
CWP: Chronic widespread pain
FM: Fibromyalgia
MNRB™: Motivational nondirective resonance breathing
VNS: Vagus nerve stimulation
tVNS: Transcutaneous vagus nerve stimulation
iVNS: Invasive vagus nerve stimulation
BP: Blood pressure
WHR: Waist-to-hip ratio
HRV: Heart rate variability
hfHRV: High-frequency heart rate variability
rMSSD: Root mean square of successive differences
lnRMSSD: Natural log of rMSSD
SDNN: Standard deviation of NN intervals
pNN50: Percentage of successive normal sinus RR intervals more than 50 ms
ECG: Electrocardiography
CPA: Computerized cuff-pressure algometry
PDT: Pain detection threshold
PTT: Pain tolerance threshold
PPL: Pressure pain limit
ANS: Autonomic nervous system
WPI: Widespread pain index
SSS: Symptom severity scale
NRS: Numerical rating scale
VAS: Visual analog scale
PPG: Photoplethysmography
PROM: Participant-reported outcome measure
eCRF: Electronic case report form
DB: Diaphragmatic breathing
